# Epigenetic distortion to VDR transcriptional regulation in prostate cancer cells

**DOI:** 10.1016/j.jsbmb.2012.10.002

**Published:** 2012-10-23

**Authors:** Prashant K. Singh, Craig L. Doig, Vineet K. Dhiman, Bryan M. Turner, Dominic J. Smiraglia, Moray J. Campbell

**Affiliations:** a Department of Pharmacology and Therapeutics, Roswell Park Cancer Institute, Buffalo, NY 14263, USA; b Institute of Biomedical Research, College of Medical and Dental Sciences, University of Birmingham, Birmingham B15 2TT, UK; c Department of Cancer Genetics, Roswell Park Cancer Institute, Buffalo, NY 14263, USA

**Keywords:** NCOR1, Prostate cancer, Epigenetics, VDR

## Abstract

The current study aimed to examine the gene specific mechanisms by which the actions of the vitamin D receptor (VDR) are distorted in prostate cancer. Transcriptional responses toward the VDR ligand, 1α,25(OH)_2_D_3_, were examined in non-malignant prostate epithelial cells (RWPE-1) and compared to the 1α,25(OH)_2_D_3_-recalcitrant prostate cancer cells (PC-3). Time resolved transcriptional studies for two VDR target genes revealed selective attenuation and repression of VDR transcriptional responses in PC-3 cells. For example, responses in PC-3 cells revealed suppressed responsiveness of *IGFBP3* and *G0S2*. Furthermore, Chromatin Immunoprecipitation (ChIP) assays revealed that suppressed transcriptional responses in PC-3 cells of *IGFBP3* and *G0S2* were associated with selective VDR-induced NCOR1 enrichment at VDR-binding regions on target-gene promoter regions. We propose that VDR inappropriately recruits co-repressors in prostate cancer cells. Subsequent direct and indirect mechanisms may induce local DNA methylation and stable transcriptional silencing. Thus a transient epigenetic process mediated by co-repressor binding, namely, the control of H3K9 acetylation, is distorted to favor a more stable epigenetic event, namely DNA methylation.

## 1. Introduction

Epigenetic mechanisms are central to the evolution of malignant phenotypes. The androgen receptor (AR) [1] exerts a profound control on the growth and differentiation of normal prostate. Its cellular actions have been studied extensively; for example the process of androgen withdrawal was exploited to define apoptosis [2]. The AR co-operates with WNT and mTOR pathways [3,4] to induce prostate epithelial cell proliferation. Equally, other nuclear receptors, such VDR, PPARs, and RARs, exert mitotic restraint, at least in part by antagonizing WNT signaling and activation of cell cycle arrest through regulation of gene targets such as *CDKN1A* (encodes p21^(waf1/cip1)^), *IGFBP3* [5–11] and *G0S2* [12,13]. In prostate cancer (CaP) the central actions of the AR are exploited in androgen deprivation therapy (ADT) to derive significant clinical benefit. Ultimately this is not sustained and treatment failure following ADT is characterized by ADT-recurrent CaP (ADT-RCaP), which is invariably lethal.

The impact of ADT on the malignant cell presents a formidable environment that cancer cells must adapt to. This process is multifaceted and includes loss of mitogenic signals downstream of the AR, triggering apoptosis, hypoxia (due to endothelial cell collapse) and inflammation that has an associated mileu of cytokine and other signals. Central aspects of the escape mechanisms to this restraint include increasing intrinsic AR ligand production and AR signaling capacity. However the transcriptional actions of the AR in ADT-RCaP are not merely a re-iteration of the normal AR transcriptome, but rather represent a fundamentally different transcriptome. Epigenetic events are central to the evolution of the altered AR signaling capacity.

The AR transcriptional program evolves toward increased targeting of proliferative gene promoters and decreased targeting of pro-differentiation genes [14,15]. For example, the oncogenic actions of the TMPRSS2/ETS fusion, a common event in CaP [16], are critical precisely because the *TMPRSS2* promoter is sustained in an AR responsive state. More recently genome-wide ChIP-chip and ChIP-Seq approaches have revealed considerable variability in the targeted transcriptional networks [17–19]. For example in CaP, as the disease progresses, there are altered levels of H3K4me1 and 2 on gene enhancer regions in the ADT-RCaP state, where cells have evolved resistance to anti-androgen therapies. In this new state the targeted increase of H3K4me1 and 2 at different enhancer regions allows the cells to initiate a different AR transcriptional program [20].

These events are not unique to CaP. In a range of solid tumors and myeloid leukemia, nuclear receptors that normally exert mitotic restraint, such as the VDR, RARs and PPARs, become skewed, with selective suppression of gene targets associated with antiproliferative actions [21–26]. Thus RARs, PPARs and the VDR display altered transcriptomes in CaP as a result of distorted epigenetic events (reviewed in Ref. [27]). Dissecting and exploiting the epigenetic mechanisms contributing to altered nuclear receptor function offer significant therapeutic promise. Therefore the development of CaP provides a key system to study the evolution of the malignant epi-genome, and defining these mechanisms is of clinical significance.

Loss and gain of function of transcriptional co-activators and co-repressors associates with transcriptional rigidity. Co-activators and co-repressors each display both loss and gain of function, and can result in similar phenotypes. Thus the loss of a co-activator can lead to suppressed ability of a transcription factor to trans-activate a given target. Similarly the gain of function of co-repressors can limit transactivation ability and enhance trans-repression. The opposite patterns will in turn enhance the trans-activation function. Compared to their co-activator cousins, the co-repressors are somewhat under-explored. Ambiguity remains over how and to what extent these actions are distorted in cancer (reviewed in Ref. [27]). The sheer diversity of transcription factors and co-repressors interactions contributes significantly to this uncertainty. This in turn is compounded by the fact that there are functionally different co-repressor isoforms [28–30] and that co-repressor actions appear specific to each phase of the cell cycle [31–33].

The proto-typical co-repressors NCOR1 and NCOR2/SMRT were cloned in 1995 using nuclear receptor as bait [34,35], and both proteins exist in large multimeric complexes (~2.0 MDa) [36] with histone deacetylases and other histone modifying enzymes (reviewed in Ref. [37]). These complexes are recruited to many different transcription factors, to repress gene activity. The importance of targeted basal repression by co-repressors is evident in the lethality of the *Ncor1*^−/−^ and *Ncor2/Smrt*^−/−^ mice [38].

Evidence has also emerged that NCOR1 and NCOR2/SMRT complexes are dynamically recruited to activated transcription factors leading to active transrepression [39], for example associated with suppression of inflammation [40]. Similarly, co-repressor induced transrepression of the glucocorticoid receptor has been established on a genome-wide scale [41]. Finally, de-repression occurs whereby loss of co-repressor association, following activated transcription factor, leads to up-regulation of target genes independently of the sustained presence of the transcription factor [42]. The first direct measurement of the genome-wide distribution of NCOR2/SMRT has established basal and activated distribution during adipogenesis and identified repression of key differentiation programs and hinted at more dynamic interactions with euchromatic regions than hitherto suspected [43,44].

Well-established oncogenic roles for NCOR1 and NCOR2/SMRT have been elucidated in acute promyelocytic leukemia that results from a fusion between RARα, and either the promyelocytic leukemia (PML) or promyelocytic leukemia zinc finger (PLZF) genes [23]. Both chimeric proteins sustain NCOR1 interactions and consequently RARα-mediated cell differentiation is blocked, in part, as a result of maintaining a condensed chromatin structure around the promoters of RARα target genes that govern normal hematopoietic differentiation [45,46]. The importance of inappropriate NCOR1 binding in these disease states has been exploited to stratify patients to tailored therapies. Furthermore the ability of steroidal nuclear receptor such as the AR and ERα to bind NCOR1 and NCOR2/SMRT is important to therapeutic exploitation with receptor antagonists in prostate and breast cancer. Therefore co-repressors appear to play roles in firstly driving critical oncogenic events, but secondly providing a rational targeted strategy toward the key histone modifying enzymes contained within the complex.

Expression profiling in solid tumors has revealed altered NCOR1 and NCOR2/SMRT expression and localization, for example in breast, bladder, and prostate cancers [21,24,26,47,48]. However, to date, uncertainty remains over their precise role in solid tumors, especially in the case of breast and prostate cancers where the etiology of disease is intimately driven by the actions of steroid nuclear receptors. In CaP cells, elevated levels of NCOR2/SMRT have been detected and suppress VDR responsiveness [21]. Similarly, PPAR actions are disrupted and can be targeted selectively by using HDAC inhibitor co-treatments [32,49]. More specifically, elevated NCOR1, and to a lesser extent NCOR2/SMRT correlated with, and functionally drove, the selective insensitivity of PPARα,γ receptors toward dietary derived and therapeutic ligands most clearly in ADT-RCaP cells [32]. Elevated levels of NCOR1 occur in ERα negative breast cancer cells and in turn attenuate anti-mitotic actions of VDR. Again, this molecular lesion can be targeted in ERα negative breast cancer cell lines with co-treatments of VDR ligand (*e.g.* 1α,25(OH)_2_D_3_) plus HDAC inhibitors resulting in selective re-expression of VDR target genes, notably *VDUP1* and *GADD45A* [24]. Together, the studies in breast and prostate cancer suggest that NR shows specificity in their interactions with co-repressors. NCOR1 appears to be involved in the regulation of receptors such as the VDR and PPARs and NCOR2/SMRT with steroid nuclear receptors; this may reflect the emergent specificities of receptor interactions observed in the murine co-repressor knockout models [50,38,51,52].

In contrast, a parallel literature has revealed loss of NCOR1 and NCOR2/SMRT is associated with the ADT-RCaP phenotype and enhances AR transcriptional programs [53,54]. Similar roles for NCOR1 and NCOR2/SMRT appear in the development of breast cancer and Tamoxifen resistance [47]. In contrast, increased NCOR1 and NCOR2/SMRT expression in CaP suppresses the responsiveness of other nuclear receptors that usually exert mitotic restraint, such as VDR and PPARα,γ [32,47–49]. Thus, in CaP progression, there are conflicting selection pressures on co-repressor expression and recruitment. To address this conflict we examined the kinetics of NCOR1 recruitment to genes that display differing transcriptional responsiveness toward 1α,25(OH)_2_D_3_ in non-malignant and malignant prostate epithelial cells.

## 2. Results

### 2.1. Altered regulation of IGFBP3 and G0S2 in prostate epithelial cells

Non-malignant and malignant prostate cell lines display a range of anti-proliferative responses toward 1α,25(OH)_2_D_3_. Non-malignant prostate epithelial RWPE-1 cell is highly responsive toward 1α,25(OH)_2_D_3_ [31] whereas the PC-3 CaP cell line, derived from a metastasis [55], is recalcitrant to the anti-proliferative actions of 1α,25(OH)_2_D_3_ [21,32,56].

**Fig. 1:**
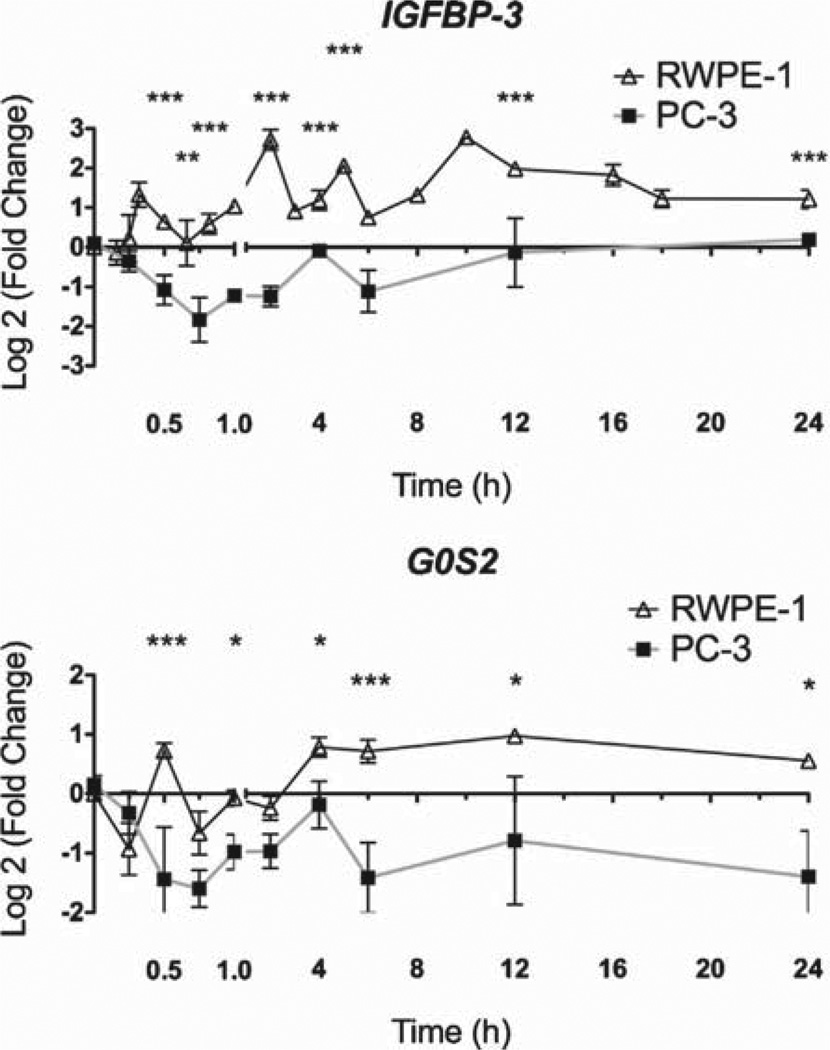
Dynamic regulation of VDR target genes. Panel A. RWPE-1 and PC-3 cells were treated with 1α,25(OH)_2_D_3_ (100 nM) or EtOH and mRNA was extracted at the indicated time points, and accumulation of *IGFBP3* and *G0S2* was measured using TaqMan Q-RT-PCR. Accumulation of each target is given as log_2_ (fold change). Each data point represented the mean of triplicate experiments in triplicate wells ±S.E.M. (*p < 0.05, **p < 0.01, ***p < 0.001).

As a functional indicator of 1α,25(OH)_2_D_3_ actions, VDR-mediated gene regulatory actions were examined in RWPE-1 and PC-3 cells. Time-resolved regulation studies were undertaken with *IGFBP3* and *G0S2*. The time-resolved kinetics in RWPE-1 and PC-3 cells for the genes are shown in Fig. 1. The kinetics of *IGFBP3* and *G0S2* mRNA regulation were highly pronounced in RWPE-1 cells. In contrast, the mRNA was significantly reduced in PC-3 at multiple time points. Together these data indicate that gene regulation by 1α,25(OH)_2_D_3_ was most dynamic in cells that were most responsive to the anti-proliferative effects (RWPE-1 cells). By comparison, in PC-3 cells, the mRNA regulation profiles were selectively attenuated.

### 2.2. Temporal distribution of NCOR1 to target genes is altered in 1α,25(OH)_2_D_3_-recalcitrant cells

**Fig. 2:**
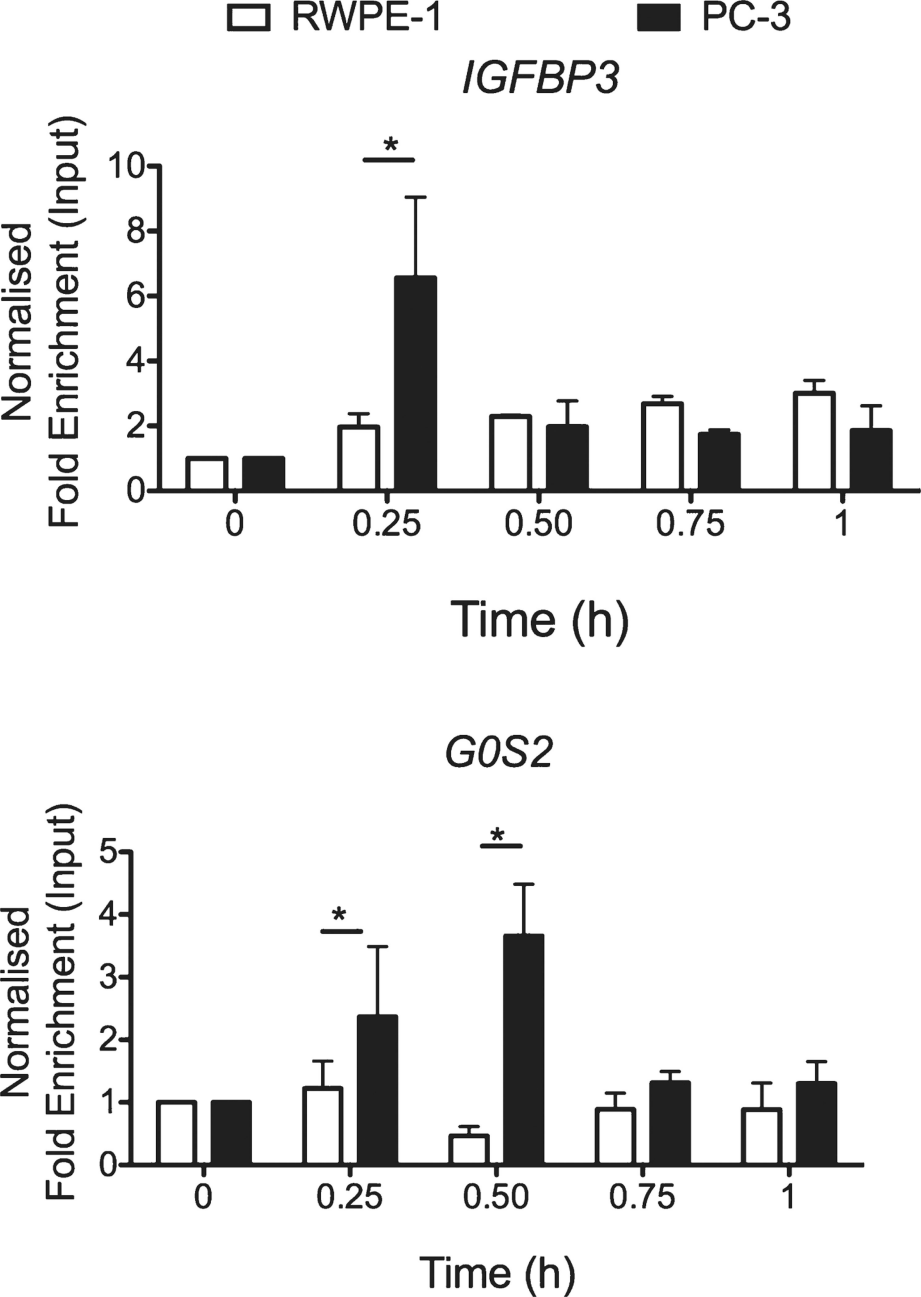
NCOR1 differentially associates with target genes. RWPE-1 and PC-3 cells were treated with 1α,25(OH)_2_D_3_ (100 nM) or EtOH for indicated time points. Association of NCOR1 was measured at each region using X-ChIP with ChIP grade antibodies and normalized, and given as fold enrichment over input [31]. Enrichment was measured using Q-PCR with primers specific to these regions that amplified products <150 bp. All measurements were performed in technical duplicate and biological triplicate (*p < 0.05, **p < 0.01, ***p < 0.001).

Q-ChIP was undertaken to examine recruitment of NCOR1 to the TSS of *G0S2* and a well-characterized VDRE on *IGFBP3* [57] in PC-3 cells compared to RWPE-1 cells (Fig. 2). Enhanced 1α,25(OH)_2_D_3_-regulated NCOR1 recruitment was evident in 1α,25(OH)_2_D_3_-recalcitrant PC-3 cells compared to 1α,25(OH)_2_D_3_-sensitive RWPE-1 at these regions examined. This occurred rapidly on both genes in PC-3 cells within the first hour of exposure to 1α,25(OH)_2_D_3_, compared to RWPE-1 cells, NCOR1 appeared to cycle off and be subsequently recruited back at later time points; at 24 h on the *IGFBP3* response element and at 2 and 12 h on the TSS of *G0S2* (data not shown). These findings suggest the underlying mechanisms of recruitment of NCOR1 in response to VDR activation differ significantly between the two cell types.

## 3. Discussion

The VDR governs and influences anti-mitotic and prodifferentiation transcriptional programs and these actions are distorted in CaP cells [10]. Therefore dissecting the 1α,25(OH)_2_D_3_-recalcitrant phenotype is of potential clinical significance. To address this aim, we examined whether the co-repressor protein NCOR1 was differentially recruited to target genes that are known to regulate these anti-mitotic transcriptional programs.

As a starting point to these questions the current study examined differential mRNA regulation of two VDR target genes in different prostate cell models. These approaches revealed that 1α,25(OH)_2_D_3_ regulated expression was attenuated and even repressed compared to vehicle controls in PC-3 cells that are recalcitrant to the anti-mitotic actions of 1α,25(OH)_2_D_3_, compared to RWPE-1 cells. Building on these studies, we examined the binding of NCOR1 following VDR activation and revealed that 1α,25(OH)_2_D_3_ induced greater NCOR1 association on the *IGFBP3* and *G0S2* promoters in PC-3 cells, compared to RWPE-1 cells. Thus NCOR1 was sustained and enriched at VDR binding sites to different extents and at different time points. Probably reflecting looping events, the TSS showed sustained NCOR1 enrichment throughout the time course [58].

It is now over 30 years since the initial reports demonstrated the anticancer actions of 1α,25(OH)_2_D_3_ [59–61]. Following these studies, anti-proliferative effects were demonstrated in a wide variety of cancer cell lines, including those from prostate [5,62–64], as well as xenograft and transgenic CaP models [65,66]. As the anti-cancer effects of the ligand emerged, large-scale epidemiological studies found inverse associations between circulating 25(OH)D_3_ and cancer risk and advanced disease [67–75]. However, while *in vitro*, *in vivo* and epidemiological data support links between replete VDR signaling, growth restraint and broad anticancer activities, clinical exploitation of this receptor has been limited. A significant impediment to translation remains the inability to predict accurately which patients will respond to either chemoprevention or chemotherapy strategies centered on vitamin D compounds. The mechanisms that drive this resistant phenotype are often illusive and probably involve multiple aspects of disruption. Key mechanisms include gene amplification of the 1α,25(OH)_2_D_3_ metabolizing enzyme CYP24A1 [76] and repression of the VDR by more general repressors such as SNAIL [77]. The process of inappropriate co-repressor recruitment leading to stable gene silencing also contributes to this phenotype and in particular may shed light on why the VDR and other nuclear receptors are often expressed in non-malignant and retained in malignant prostate epithelial cells [32].

The process of inappropriate co-repressor recruitment may lead to stable gene silencing and in turn shed light on why the VDR and other nuclear receptors are expressed in non-malignant and retained in malignant prostate epithelial cells seemingly independent of the antiproliferative response [32].

The differential recruitment of co-repressors also addresses another ambiguity in their cancer biology, namely the impact of altered expression of co-repressors. Increased NCOR1 and NCOR2/SMRT expression occurs also in breast and bladder cancer and suppressed the responsiveness of nuclear receptors that exert mitotic restraint, such as VDR and PPARα,γ [21,24,26,32,47–49,78]. In contrast, other studies have shown that down-regulated NCOR1 and NCOR2/SMRT enhanced AR transcriptional programs in CaP [53,54,79]. The current study suggests that distorted co-repressor recruitment may provide a route for the selective silencing of critical transcriptional programs and thereby allow CaP cells to escape VDR-regulated mitotic restraint.

There is compelling evidence that histone and DNA methylation processes disrupt transcriptional actions, both alone and together. For example, one consequence of NCOR1 and NCOR2/SMRT association at target genes is the loss of H3K9ac and accumulation of H3K9me2, allowing the potential for hypermethylation at adjacent CpG regions. Further links exist between NCOR1 and DNA methylation through its interaction with KAISO [80]. Similarly regions of H3K9 and −K27 methylation, have the potential to recruit heterochromatin binding protein 1 (HP1) [81]. The recruitment of HP1 in turn re-enforces the recruitment of H3K9 methylase (KMT1A/SUV39H1) [82] and DNA methyltransferases (DNMTs) [83]; enzymes that add and sustain repressive methylation marks to histones and CpG (reviewed in Ref. [84]). Thus the processes of repressive histone modifications and DNA methylation become self-reinforcing. To complement stable DNA methylation of genomic regions, transcription by nuclear receptors appears to be associated with dynamic changes in DNA methylation [85–87]. It therefore seems possible that one consequence of increased co-repressor recruitment to target genes is the loss of H3K9ac and the accumulation of H3K9me2 that may facilitate hypermethylation at adjacent CpG regions. Thus co-repressor actions can direct DNA methylation either through the histone modifications that they drive or through the physical association of DNA methyltransferases. In this manner the inappropriate recruitment of co-repressors may attract the DNA methylation machinery and drive selective and stable gene silencing. It is interesting to note also that the VDR has also been shown to regulate specific histone demethylases [88] adding a further layer of complexity to these relationships and the specificity of gene locus targeting.

## 4. Materials and methods

### 4.1. Agents

1α,25(OH)_2_D_3_ (gift of Dr. Milan Uskokovic, BioXell S.p.A., Italy) stored as 1 mM stocks in ethanol.

### 4.2. Q-RT-PCR

RNA was isolated using TRizol (Invitrogen). Target gene expression was quantitated on an ABI 7900 (Applied Biosystems^®^) machine using TaqMan assays. Measurements were performed in technical and biological triplicate. The statistical significance was calculated using Student’s *t*-test.

### 4.3. ChIP protocols

X-ChIP was used to measure the association of NCOR1 binding as described previously [31]. Briefly, chromatin from 1.5 × 10^6^ mid-exponential cells was cross-linked. Pre-cleared inputs were immunoprecipited with NCOR1 (Abcam ab24552). Complexes were recovered using magnetic beads, washed, cross-linking was reversed and further cleared DNA was recovered by standard precipitation approaches. 25 ng DNA was used per Q-PCR reaction using SYBRgreen with pre-optimized primers as described previously [31]. Student’s *t*-test was used to calculate the significant enrichment.
